# Public Mental Health: Global Perspectives

**DOI:** 10.1192/pb.bp.113.045484

**Published:** 2014-12

**Authors:** Oyinlola Oyebode

This book aims to give an overview of the key issues in public mental health. It is timely as many populations are suffering the depressing consequences of the economic downturn, interest in mental health and well-being by policy makers is increasing, and the recent World Health Organization’s Mental Health Action Plan moves this issue to the top of the health agenda.

The book is structured in four parts examining the promotion of mental health and well-being; the prevention of mental health problems; enhancing the lives of people with mental health problems; and finally, bringing these three lines of action together to explore public mental health at each life stage.

This is an anthology of essays. Each chapter is written by an expert or experts from their individual professional and political stance. Do not expect to be convinced of one specific set of actions which must be taken to improve public mental health; instead, the book encourages reflection on the variety of possible approaches, allowing the reader to consider these viewpoints and make their own informed decision about what they might champion in their own area.

The chapter by Wilkinson & Pickett, singled out as a highlight on the back cover blurb, will not offer much that is new if you have read their book *The Spirit Level* (or a good summary); however, it is good to revisit this in the context of very different perspectives. I most enjoyed the chapters on measuring mental health and on suicide prevention which were thought-provoking and absorbing enough to read on a homebound train after a long day.

My main criticism of this book is that although it claims to contain global perspectives, and there are many internationally renowned writers, it is heavily dominated by the UK setting, with most international references being to other English-speaking countries. For example, of the 17 case studies, 11 were UK based and 2 were from Australia and New Zealand. This did not detract from the book’s utility for me, as a UK-based practitioner, but could disappoint others.

The only other warning I would give is that the book does not start from a basic level, for example assuming knowledge of Geoffrey Rose’s work. If you are motivated to learn about public mental health, get a grounding in public health first, then buy this book.

